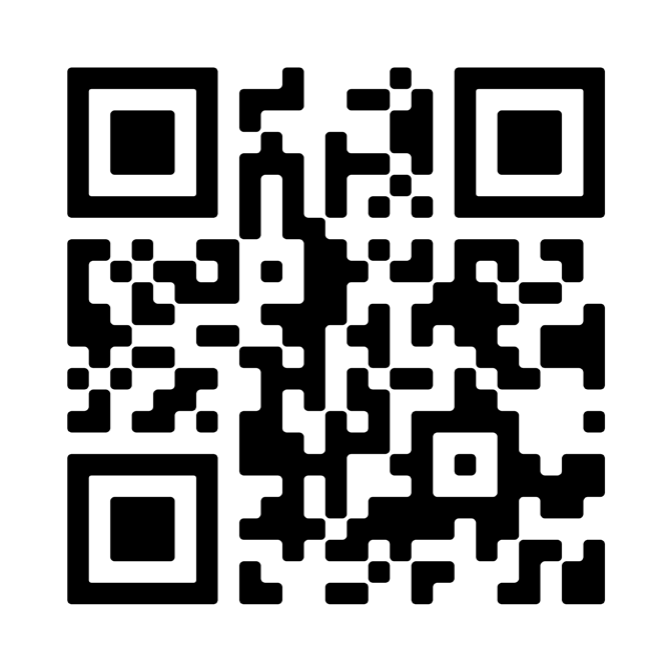# HUGO(TM) RAS System in urogynaecology: the first nerve sparing Sacral Colpopexy for Pelvic Organ Prolapse

**DOI:** 10.52054/FVVO.15.1.054

**Published:** 2023-03-31

**Authors:** G Panico, G Campagna, D Caramazza, L Vacca, S Mastrovito, A Ercoli, G Scambia

**Affiliations:** Fondazione Policlinico Universitario A. Gemelli IRCCS, UOC Chirurgia Ginecologica, Dipartimento di Scienze della Salute della Donna e del Bambino e di Sanità Pubblica, Università Cattolica del Sacro Cuore, Roma, Italia; PID Ginecologia Oncologica e Chirurgia Ginecologica Miniinvasiva, Università degli studi di Messina, Policlinico G.Martino, Messina, Italia.

**Keywords:** Laparoscopy, pelvic organ prolapse, colposacropexy, anatomy, dissection

## Abstract

**Background:**

Minimally invasive sacral colpopexy is considered the gold standard for surgical treatment of Pelvic Organ Prolapse (POP), combining high success rates with low recurrence risk in comparison to other techniques. This is the first case of robotic sacral colpopexy (RSCP) performed with the innovative Hugo™ RAS robotic system.

**Objectives:**

The aim of this article is to show the surgical steps of a nerve sparing RSCP performed with the new Hugo™ RAS robotic system (Medtronic), by also evaluating the feasibility of this technique using this novel Robotic System.

**Materials and Methods:**

A 50-year-old Caucasian woman with symptomatic pelvic organ prolapse (POP-Q): Aa: +2, Ba: +3, C: +4, D: +4, Bp: -2, Ap: -2 , TVL:10 GH: 3,5 BP:3 underwent RSCP as well as a subtotal hysterectomy with bilateral salpingo-oophorectomy, using the new surgical robot Hugo™ RAS in the Division of Urogynaecology and Pelvic Reconstructive Surgery, Fondazione Policlinico Universitario A. Gemelli IRCCS, Rome, Italy.

**Main Outcome Measures:**

Intraoperative data, docking specifics, objective and subjective outcomes at three months follow up.

**Results:**

Surgical procedure was carried out without intra-operative complications, operative time (OT) was 150 minutes, docking time was 9 minutes. No system errors or faults in the robotic arms were registered. Urogynaecological examination at three months follow up showed a complete resolution of the prolapse.

**Conclusion:**

RSCP using the Hugo™ RAS system seems to be a feasible and effective approach according to results in terms of operative time, cosmetic results, postoperative pain and length of hospitalisation. Large number of case reports as well as longer follow up are mandatory to better define its benefits, advantages, and costs.

## Learning Objective

Hugo™ RAS System (Medtronic, Minneapolis, MN, USA) is a novel robotic technology recently introduced in Gynaecologic and Urologic surgery (Ragavan et al., 2022; Monterossi et al., 2022). Evaluation of robotic tools and surgical skill has become increasingly important since robotic approaches to common surgeries become more widely utilised. This video shows the surgical steps of Robotic nerve sparing Sacral Colpopexy procedure underlining its feasibility using the new Hugo™ RAS robotic system.

## Introduction

Pelvic Organ Prolapse (POP) is defined as the descent of one or more of the anterior vaginal wall, posterior vaginal wall, uterus (cervix) or vaginal vault (cuff scar after hysterectomy) (Haylen et al., 2016). It is a common condition negatively affecting the quality of life of a high percentage of women (Brown et al., 2022).

Minimally invasive Sacral Colpopexy is nowadays considered the gold standard for the surgical treatment of advanced POP, combining high success rates with low recurrence risk compared to other techniques (Maher et al., 2016).

Minimally invasive surgery (MIS) which aims to reduce invasive operations, is increasing in strength over the years as it continues to reduce hospitalisation and improved recovery for patients.

After the Food and Drug Administration’s (FDA) approval in 2005 and the successive introduction of the first robotic systems, MIS has had major improvements in terms of surgical learning curve and feasibility of many surgical procedures across the world. Approved indications include management of most benign and malignant diseases in urology and gynaecology (Capozzi et al., 2022).

The most widely available platform with extensive installations is the DaVinci® robotic Surgical System (Intuitive Surgical Inc.) In recent years, other robotic platforms have emerged. Our group also previously published studies regarding the use of ALF-X by Senhance (TRANSENTERIX Inc., USA) to perform RSCP (Panico et al., 2020).

Hugo™ RAS represents the newest alternative to traditional robotic systems. It is characterised by independent Arm carts, which can be used with a three or four-arm configurations depending on the procedure, a system tower, and an open console.

This is the first case of robotic sacral colpopexy (RSCP) performed with the innovative Hugo™ RAS robotic system.

## Patients and methods

A 50-year-old woman affected by symptomatic POP was referred to our Urogynaecological Division of Fondazione Policlinico Universitario A. Gemelli IRCCS, Rome, Italy, and underwent nerve-sparing RSCP. She was Caucasian with a body mass index (BMI) of 22 Kg/m2 and was affected by symptomatic POP (POP-Q Aa: +2, Ba: +3, C: +4, D: +4, Bp: -2, Ap: -2, TVL:10 GH: 3,5 BP:3).

For routine pre-operative clinical work up, existing medical history, physical examination, POP-Q scores evaluation, laboratory exams, pelvic and urinary tract ultrasound, and a urodynamic examination were performed. The patient reported two normal vaginal deliveries with no complications, no previous surgical procedures, and she was in menopause since the age of 45.

She complained of frequency, vaginal bulging, sense of incomplete bladder emptying, hesitancy but without stress urinary incontinence. The ultrasound evaluation revealed no hydronephrosis, normal uterus, ovaries, bladder, and kidneys; Pap test was normal.

At urodynamic evaluation performed after manual prolapse reduction, the filling phase appeared normal, as there was no evidence of urodynamic stress incontinence or detrusor overactivity, and compliance was normal. The emptying phase was carried out with abdominal muscles use, suggesting a bladder neck obstruction associated to a reduced maximum flow rate (Q max: 9ml/s) with a normal voiding detrusor pressure. No post voiding residual urine was noted.

The patient was informed about conservative options but declined the use of pessaries. As she was post-menopausal, the patient refused uterine sparing techniques and was given the option of undergoing a subtotal hysterectomy and bilateral salpingo-oophorectomy associated to RSCP.

Prior to surgery the patient received an accurate surgical counselling, she was given information on different surgical approaches (laparoscopic, abdominal, and vaginal approaches, native tissue repair), was advised about risks of the procedure and signed an informed consent allowing the use of personal data.

## Results

**Figure 1:**
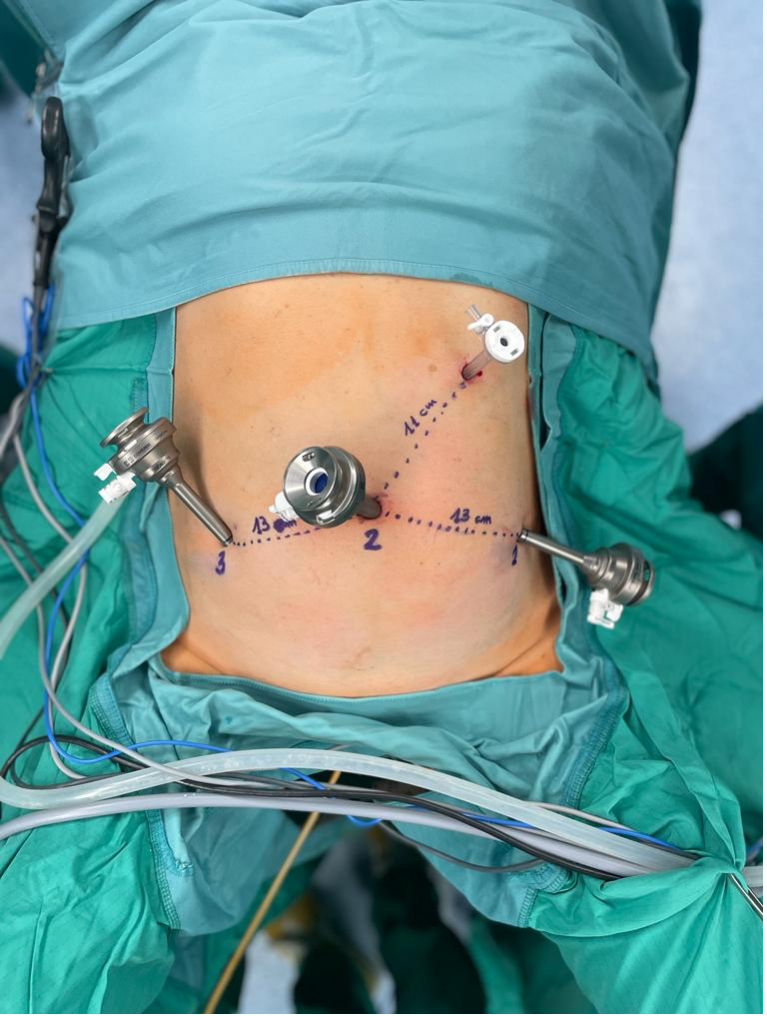
Trocar positioning.

The procedure started by inserting a 12-mm optic port in umbilical position. Once pneumoperitoneum at 12 mmHg was reached, a 3D-HD 0° 10 mm scope (Karl Storz Endoscopy) was inserted. Two additional 8 mm ports were placed under direct visualisation in the right and left lower abdomen, at 13 cm distance from the umbilical port and 5 cm below the trans umbilical plane. An additional 5-mm trocar was placed at palmer’s point, for the first assistant’s use (Figure 1).

Patient positioning, pre and post operatory prophylaxis and the procedure were carried out using a standardised technique, as already published by our group (Campagna et al., 2018; Panico et al., 2021). The surgical steps are shown in detail in the attached video.

**Figure 2:**
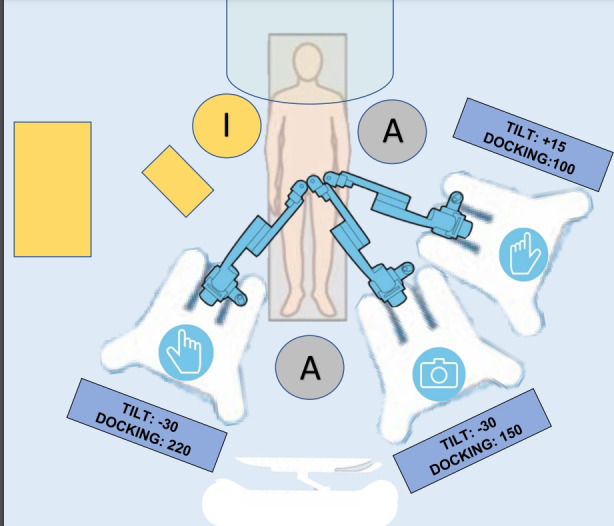
Overview of the operating room.

A three robotic arms configuration was chosen, and assistant surgeons hooked the robotic arms to the trocars. The robotic instruments used were a bipolar grasper, monopolar scissors and two needle holders. Different graspers, metallic clip applicator and suction irrigation cannula were used by the first assistant surgeon through the 5mm port. During the procedures, the second assistant surgeon moved the uterus using a uterine manipulator. An overview of the operating room arrangement is shown in Figure 2.

The instrument movement scale was 1.5:1 and 2x respectively for speed and rotation.

Two adequately shaped polypropylene mesh, Restorelle (Coloplast, Minneapolis, MN) were chosen to fix to the anterior and posterior vaginal walls through multifilament non absorbable sutures, Ethibond (Ethicon, Inc., Somerville, NJ).

Total operative time (defined as the interval from the start of procedure to the suture of surgical incisions) was 150 minutes. Total docking time (defined as the time to adapt the robotic setting to the patient, to move the robotic arms and the scope) was 9 minutes. Console time (the interval from the moment the first operator started the procedure from the console, until the end of its usage) was 120 minutes. Estimated blood loss was 30mL. No complications were noted according to Dindo classification (Dindo et al., 2004). Pain VAS score was 4-4-2-2 respectively at 2,4,12 and 24 hours after surgery (McCormack et al., 1988). Patient was discharged on the second postoperative day. The postoperative urogynaecological examination at discharge demonstrated a complete resolution of the prolapse, and the satisfaction value regarding subjective outcome through Patient Global Impression of Improvement (PGI-I) was excellent (Srikrishna et al., 2010).

At three months follow up the urogynaecology examinations confirmed the postoperative anatomical outcome with apex well suspended and vaginal wall perfectly lifted. (POP-Q) Aa: -3, Ba: -3, C: -8, D: -8.5, Bp: -3, Ap: -3, TVL:10 GH: 3,5 BP:3).

No urinary symptoms were complained, supporting the improvement in POP related symptoms already referred during patient’s interview.

## Discussion

To our knowledge, this is the first RSCP procedure performed with the new Hugo™ RAS robotic system.

Since its introduction, robotic surgery gained popularity across the world due to its many advantages for both surgeons and patients. A recent meta-analysis comparing gynaecologic laparoscopic procedures with and without robotic assistance, showed a shorter hospital stay and less intraoperative blood loss in the robotic group (Marchand et al., 2021). Furthermore, increased accuracy, faster suturing, and reduced number of errors seem to be among the many advantages of Robotic-assisted laparoscopy over conventional laparoscopy, with little or no difference in complication rates (Lawrie et al., 2019). Robotic Assisted Surgery allows three-dimensional view, magnified vision, articulating wrists allowing multiaxial movement, lack of hand tremor, and surgeon comfort. On the other hand, there still are some specific limitations, such as the absence of tactile feedback and the high costs compared to conventional laparoscopy (Pan et al., 2016).

More specifically the Hugo™ RAS robotic system seems to be a promising technology since it includes many advantages of the systems already in use, adding some potential benefits due to its advanced technical details: the independent arms give free access to the patient from different angles. Furthermore, the open console and eye tracking system allow the surgeon to be completely aware of his surroundings in the operating room.

In addition, the trocar positioning in the Hugo™ RAS robotic system is more versatile thanks to the independent arms, which allow the surgeons to opt for a similar setting to the one used in a standard laparoscopic sacral colpopexy, which is relevant since it would give the surgeon the possibility of a convenient and fast conversion to standard laparoscopic setting if in need.

The operative time for this particular procedure was comparable to standard laparoscopic sacral colpopexy, but comparatively shorter than timings described in literature for RSCP (Pan et al., 2016; Chang et al., 2021; Chang et al., 2022).

During the procedure, there was no need to relocate any robotic arm due to limiting position or arm collision.

The instrument movement scale of 1.5:1 and 2x respectively for speed and rotation combined with the specifically designed handles permitted a safe manipulation of tissue and easy suturing.

Although this trocar positioning setting seems to be favourable in terms of freedom of movements of the robotic arms, the first surgeon was forced to only use the lateral operative arms during the procedure, which differed from standard laparoscopy where surgeon would have been able to use a median suprapubic port as well. Although this was not an issue in this case, this could represent a technical challenge in case of a particularly challenging retroperitoneal pelvic dissection.

## Conclusions

In conclusion, RSCP performed using the Hugo™ RAS system seems to be a feasible and effective approach with good results in terms of operative time, intraoperative blood loss, postoperative pain, length of hospitalisation, anatomical results, and patient’s satisfaction. Our case report may represent the basis of future studies to confirm the safety, efficacy, and feasibility of the technique. Large series of case reports as well as a longer follow up are without doubt needed to better define the advantages or possible disadvantages of this novel approach.

## Video scan (read QR)

https://vimeo.com/748045071/765828d1cb

**Figure:**